# Role of mTOR in Glucose and Lipid Metabolism

**DOI:** 10.3390/ijms19072043

**Published:** 2018-07-13

**Authors:** Zhuo Mao, Weizhen Zhang

**Affiliations:** 1 Center for Diabetes, Obesity and Metabolism, Department of Physiology, Shenzhen University Health Science Center, Shenzhen 518060, China; maoz@szu.edu.cn; 2 Department of Physiology and Pathophysiology, Peking University Health Science Center, Beijing 100191, China

**Keywords:** mTOR, metabolic diseases, glucose and lipid metabolism

## Abstract

The mammalian target of rapamycin, mTOR is the master regulator of a cell’s growth and metabolic state in response to nutrients, growth factors and many extracellular cues. Its dysregulation leads to a number of metabolic pathological conditions, including obesity and type 2 diabetes. Here, we review recent findings on the role of mTOR in major metabolic organs, such as adipose tissues, liver, muscle, pancreas and brain. And their potentials as the mTOR related pharmacological targets will be also discussed.

Multicellular organisms evolve essential mechanisms to sense and accommodate the ever-changing extracellular environments for their survival and growth. The mechanistic target of rapamycin (mTOR) signaling is the most important intracellular pathway that coordinates local nutrients and systemic energy status at the organismal and cellular level. Dysregulation in mTOR signaling is associated with various diseases such as obesity, type 2 diabetes, cancer, and neurological diseases [1]. Obesity and over-nutrition induce a chronic hyper-activation of mTOR activity in multiple tissues [2,3,4]. In turn, mTOR signaling dysregulation may facilitate the development of type 2 diabetes mellitus (T2DM) or insulin resistance. In this review, we provide a comprehensive summary on the mTOR signaling in the regulation of glucose and lipid metabolism. We will focus on the recent findings about the role of mTOR complex (mTORC) pathways in the regulation of energy balance and metabolism in key metabolic tissues, including adipose tissue, liver, skeletal muscle, pancreas and the brain. We will also briefly discuss the therapeutic potential of mTOR signaling for the metabolic disorders.

## 1. mTOR Signaling 

**Figure 1:**
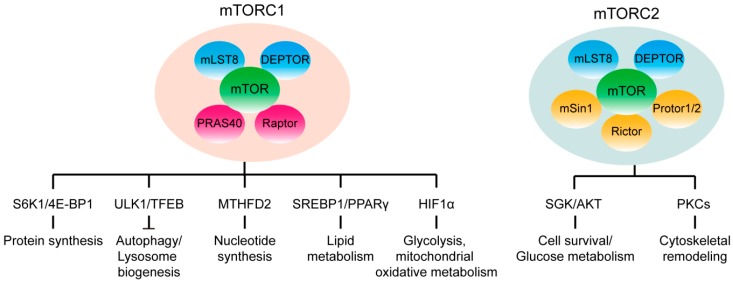
The protein components and the major downstream signaling pathway of mTORC1 and mTORC2. Simplified illustration of the protein components of mTORC1 and mTORC2 complex. Activation of mTORC1 promotes protein synthesis, nucleotide synthesis, lipogenesis, glycolysis, and inhibits autophagy and lysosome biogenesis. Alternatively, mTORC2 regulates cell survival/glucose metabolism and cytoskeletal remodeling.

mTOR is the conserved serine/threonine kinase which exists in two distinct multi-complexes with different protein components and downstream substrates: mTOR complex 1 (mTORC1) and mTOR complex 2 (mTORC2) (Figure 1). Both two complexes shared some common protein components: mTOR (a serine/threonine protein kinase), mLST8 (mammalian lethal with sec-13 protein 8) and DEPTOR (DEP-domain containing mTOR-interacting protein). The additional mTORC1 complex proteins include scaffold protein Raptor (regulatory-associated protein of TOR) and Akt substrate protein PRAS40 (proline-rich Akt substrate 40 kDa). The mTORC2 core components include scaffold protein Rictor (rapamycin insensitive companion of mTOR), mSIN1 (stress-activated protein kinase-interacting protein 1), and protein observed with rictor 1 and 2 (PROTOR1/2) [1,3].

mTORC1 mainly maintains a cellular balance between anabolism and catabolism in response to the environmental cues such as growth factors, amino acids, and stress. Growth factors such as insulin and insulin-like growth factor (IGF) regulate mTORC1 through phosphoinositide 3-kinase (PI3K)-AKT-Tuberous sclerosis (TSC)-RHEB signaling. PI3K-AKT signaling phosphorylates, and inhibits TSC1 which is the GTPase-activating protein (GAP) for the RHEB (the small GTPase Ras homologue enriched in brain), thus activating RHEB. mTORC1 activity is strongly enhanced by active RHEB. Amino acids activate mTORC signaling by stimulating the Rag family of GTPases, which promotes mTORC1 translocation to the lysosome and is activated by RHEB [5,6]. Stress such as hypoxia, DNA damage also signals to mTORC with multiple mechanisms while in general through TSC1/2 [3,7].

Activation of mTORC1 induces protein synthesis by promoting ribosome biogenesis and mRNA translation. The major downstream effectors of mTORC1 are the ribosomal S6 kinase (S6K) and the inhibitory eIF4E-binding proteins (4E-BPs). Activation of S6K by mTORC1 phosphorylates its downstream substrates such as ribosomal protein S6, protein synthesis initiation factor 4B (eIF4B), and elongation factor 2 kinase (eEF2K), which subsequently promote translation initiation and elongation. Phosphorylation of 4E-BP by mTORC1 dissociates its binding with the eukaryotic translation initiation factor 4F (eIF4F), promoting 5′ cap-dependent translation [1,8]. 

In addition to protein synthesis, activation of mTORC1 is sufficient to stimulate other metabolic pathways. For example, mTORC1 enhances nucleotide synthesis by increasing ATF-dependent expression of MTHFD2, the key enzyme in mitochondrial tetrahydrofolate (mTHF) cycle, increasing the production of purine nucleotides [9]. mTORC1 promotes de novo lipogenesis in SREBP1 dependent pathway either through S6K1 phosphorylation [10] or by modulating the Lipin 1 localization and SREBP1 expression [11]. mTORC1 also stimulates glycolysis and glucose uptake through modulating the transcription factor hypoxia-inducible factor (HIF1α) [10]. 

The autophagy-lysosome and ubiquitin-proteasome are two major pathways for protein and organelle turnover. mTORC1 is implicated in these two routes to affect protein degradation. ULK1 is the mammalian autophagy-initiating kinase which drives autophagosome formation. Under nutrient sufficient condition, mTORC1 phosphorylates ULK1 and prevents its activation by adenosine 5′-monophosphate (AMP)-activated protein kinase (AMPK), blocking autophagy induction [12]. mTORC1 activation suppresses lysosome pathway through inhibiting the activity of the master regulator of lysosomal biogenesis, transcription factor EB (TFEB). Nutrient deprivation or inhibition of mTORC1 activates TFEB by promoting its nuclear translocation, thus initiating the expression of lysosomal and autophagic genes [13]. Recently, two reports have demonstrated that the ubiquitin proteasome system (UPS) in mammalian cells is increased when mTORC1 signaling pathway is inactivated [14,15]. Therefore, mTORC1 activation induces a coordinated response between lysosomal and proteasomal degradation in order to meet the rising needs of cells. 

Relative to mTORC1, the upstream signals and downstream substrates of mTORC2 are less known. mTORC2 can be activated by the growth factors such as insulin and IGF, but insensitive to the nutrients. Activated mTORC2 phosphorylates AGC kinase family, including AKT, SGK, and PKCα, to regulate cellular survival and metabolism, as well as cytoskeletal remodeling. The most well characterized substrate of mTORC2 is AKT which is phosphorylated at the serine 473. AKT could further phosphorylate TSC2, the upstream inhibitor of mTORC1. Therefore, activation of mTORC2 inactivates mTORC1. Vice versa, mTORC1-S6K axis could also directly phosphorylate mSIN1, the core component of mTORC2 and inactivate it. Therefore, mTORC1 and mTORC2 form a feedback loop regulating the complex activity. 

## 2. mTOR Signaling in Adipose Tissue

Fat or adipose tissue is a critical organ in the development of obesity and insulin resistance. mTORC signaling has been involved in adipose tissue biology in multiple aspects. mTOR is critical for adipogenesis and maintenance of fat tissues. Adipocyte-specific mTOR knockout mice have reduced adipose tissue mass, insulin resistance and fatty liver, suggesting its critical role in adipogenesis and systemic energy metabolism [16]. The role of mTORC1 complex in adipose tissue has been examined using different transgenic mouse models, including genetic depletion of S6K, S6K1, and Raptor in systemic or adipose tissue specific manner. These models consistently showed that ablation of mTORC1 signaling induces reduced adipose tissue mass and resistance to diet-induced obesity. Recent finding has identified glutamylprolyl-tRNA synthetase (EPRS) as the downstream effector of mTORC1-S6K1 axis for adiposity. Activation of mTORC1-S6K1 phosphorylates EPRS at Ser999 and induces its release from the amino acyl tRNA multisynthetase complex. Phosphorylated EPRS interacts with fatty acid transport protein 1 (FATP1), promoting its translocation to the plasma membrane and importing fatty acid to the cells [17]. 

There are two major adipose tissues, white adipose tissue (WAT) and brown adipose tissue (BAT). WAT stores energy in the form of triglyceride droplets and BAT dissipates energy through uncoupled respiration and heat production. WAT is able to acquire brown fat characteristics, a process named browning or beigeing, which is an important physiological response to cold or stress. mTORC1 is involved in the conversion of these two adipose tissues. In 2015, Xiang et al. found that activation of mTORC1 in adipose tissue increases lipid accumulation in BAT which is associated with down-regulation of brown adipocyte markers and concurrent up-regulation of WAT markers. These observations indicate a phenotypic switch of BAT to WAT. Rapamycin treatment reverses this process in vivo and in cultured brown adipocytes [18]. Later, another study found that WAT browning stimulated by catecholamine also requires mTORC1 and Raptor. Catecholamine stimulates β3 adrenergic receptor mediated cAMP-PKA signaling. PKA phosphorylates mTOR and Raptor, thus initializes browning of WAT via mTORC1-S6K1 axis. Mice with genetic deletion of Raptor or treated with rapamycin are cold intolerant with decreased browning/beigeing ability [19,20]. 

The role of mTORC2 in adipose tissue has also been examined using adipose-specific deletion of mTORC2 core component Rictor in mice. These transgenic mice have increased body size and enlarged organs, such as pancreas and heart, indicating a role of adipose mTORC2 in controlling whole body growth [21]. In addition, Rictor-null adipose cells are unable to suppress lipolysis in response to insulin, leading to elevated circulating fatty acids and glycerol [22]. mTORC2 promotes the phosphorylation of the BSD domain containing signal transducer and Akt interactor (BSTA) and its interaction with Akt1. BSTA-Akt1 interaction suppresses the expression of FoxC2, the transcription factor critical for adipocyte differentiation [23]. Moreover, mTORC2 in adipose tissue promotes de novo lipogenesis and hepatic glucose metabolism through increasing the expression of the lipogenic transcription factor ChREBPβ [24]. The role of mTORC2 in BAT growth was examined using Myf5-Cre expressed BAT precursor cells. Rictor deficiency blocks the BAT differentiation and shifts BAT metabolism to a more oxidative and less lipogenic state and protects mice from obesity and metabolic disorders [25]. mTORC2 is also implicated in WAT browning process. β-adrenergic stimulation activates mTORC2 and stimulates Akt-mediated glucose uptake and glycolysis. Loss of mTORC2 in BAT leads to cold intolerance due to defective insulin stimulated glucose uptake [26].

In conclusion, mTORC1 participates in normal adipose tissue growth and BAT-WAT phenotypic switch. mTORC2 regulates fat cell and whole body organ size, systemic glucose and lipid metabolism and BAT differentiation.

## 3. mTOR Signaling in Liver

The liver is a critical organ for systemic metabolism. In the fasted state, the liver increases ketone body production (ketogenesis), providing energy sources for peripheral tissues. mTORC1 controls ketogenesis in mice in response to fasting. Hyperactivation of mTORC1 in liver leads to a pronounced defect in ketone body production and a fasting-resistant increase in liver size. PPARα (peroxisome proliferator activated receptor α) is the master transcriptional activator of ketongenic genes which is induced by fasting. Inhibition of mTOR is required for this process. Activation of mTORC1 suppresses PPARα activity and thus the ketone production [27]. 

In addition, activation of mTORC1 signaling stimulates de novo lipogenesis in hepatocytes [28]. mTORC1 regulates hepatic lipid metabolism mainly through SREBP1, the master regulator of lipid synthesis. It is initially synthesized as an inactive precursor and localized in the ER. In response to the insulin signaling, SREBP1 is cleaved and transported to the nucleus to induce lipogenic gene expression. Liver-specific inhibition of mTORC1 abrogates SREBP1 function and renders mice resistant to hepatic steatosis and hypercholesterolemia induced by the Western diet. In 2011, Peterson et al. found that mTORC1 regulates SREBP1 through controlling the nuclear entry of a phosphatidic acid phosphatase, Lipin 1. Normally, dephosphorylated Lipin1 traffics to the nucleus and inhibits SREBP transcriptional activity and SREBP protein abundance. mTORC1 could phosphorylate Lipin 1, preventing its translocation to nucleus and hence promoting SREBP1-mediated lipogenesis [11]. Later, Han et al. demonstrated that mTORC1 also regulates SREBP1’s trafficking and maturation through CREB regulated transcription coactivator 2 (CRTC2). CRTC2 disrupts COPII dependent SREBP1 trafficking by competing with Sec23A for the interaction with Sec31A, and thus inhibits SREBP1’s maturation and function. In the feeding state or under the insulin signaling, mTOR activation phosphorylates and attenuates its inhibitory effect on SREBP1 maturation, thus enhancing lipogenesis [29]. 

Raptor is the important component of mTORC1 signaling pathway. Raptor deficient mice or cellular models have been used to study the biological functions of mTORC1 inactivation. Recently, Kim et al. have found that there are two forms of Raptor in cells, the mTORC1-bound and the free state. Although mTORC1-Raptor promotes lipogenesis through SREBP1 as previously discussed, free Raptor could increase the Akt phosphatase PHLPP2 level and decrease hepatic Akt activity, thus suppressing lipogenesis. It is proposed that the balance between free and mTORC1-bound Raptor is an important modulation mechanism for hepatic lipid accumulation [30].

In addition, activation of mTORC1 regulates whole-body behavior and metabolism. Liver-specific Tsc1 knockout mice have reduced level of hepatic and plasma glutamine, leading to peroxisome proliferator—activated receptor γ coactivator-1α (PGC-1α)—dependent fibroblast growth factor 21 (FGF21) expression in the liver. FGF21 significantly impacts the locomotor activity, body temperature, and hepatic lipid content [31]. 

The Sestrins are a family of stress-inducible proteins which suppress mTORC1 signaling activity through activation of AMPK. There are three Sestrins, named Sestrin 1, Sestrin 2, and Sestrin 3 in mammals. Hepatic mTORC1 signaling is regulated by Sestrin2. Over-nutrition and obesity induces hepatic Sestrin 2 expression primarily through activation of ER stress signaling. Increased Sestrin 2 potentiates AMPK activation and suppresses mTORC1-S6K activity in the liver, alleviating insulin resistance and obesity associated nonalcoholic fatty liver disease (NAFLD) pathologies including steatohepatitis and hepatic fibrosis. Loss of Sestrin 2 in mice displayed hyperactivation of mTORC1-S6K signaling in the liver and leads to insulin resistance and glucose intolerance when fed with high fat diet [32,33]. In addition to Sestrin 2, metformin, the most widely used drug for T2DM patients, also regulates mTORC1 activity. Recently it is found that metformin robustly inhibits mTORC1 activity and protein synthesis in liver. This inhibition is dependent on AMPK and TSC complex [34]. 

Hepatic mTORC2 regulates glucose and lipid metabolism via AKT signaling. The role of hepatic mTORC2 has been examined in vivo using the mice lacking Rictor in liver. Deficient expression of mTORC2 in liver leads to defective insulin-stimulated AKT phosphorylation, resulting in constitutive gluconeogenesis, impaired glycolysis and lipogenesis by altering hepatic glucokinase and SREBP1c activity [35,36]. In addition, mTORC2 has been known to regulate gluconeogenesis and lipogenesis through a number of transcription factors including FOXO1, FOXA2, and PPARγ. Genomic and phosphoproteomic analyses have shown that hepatic mTORC2 regulates a complex genetic expression which affects intermediary metabolism, ribosomal biogenesis, and proteasomal biogenesis. These findings suggest that hepatic mTORC2 exerts broad biological effects under physiological conditions [37]. Similar to Sestrin 2, Sestrin 3 is an upstream regulator of mTORC2 activity. Sestrin 3 interacts with Rictor to activate mTORC2 and AKT. Therefore, deletion of Sestrin 3 in the liver results in insulin resistance and glucose intolerance and Sestrin 3 transgenic mice are protected against insulin resistance induced by a high-fat diet [38]. 

mTORC1 and mTORC2 can be regulated by an upstream regulator Reptin, an AAA + ATPase that is overexpressed in hepatocellular carcinoma. In normal adult liver, Reptin exerts opposite regulation on mTORC1 and mTORC2. It activates mTORC1 activity while inactivates mTORC2 activity, thus regulating global glucido-lipidic homeostasis. Liver-specific ablation of Reptin strongly inhibits hepatic mTORC1 activity, leading to significant decrease in *de novo* lipogenesis and cholesterol production. Meanwhile, mTORC2 activity is greatly enhanced and hepatic glucose production is inhibited [39]. 

## 4. mTOR Signaling in Muscle

Skeletal muscle tissues comprise 40% of the total body lean mass and contributes to the regulation of whole-body metabolism in various ways. It is the main organ responsible for insulin-induced glucose uptake. Insulin resistance in skeletal muscle is the primary defect during the development of type 2 diabetes. The complex role of mTOR activity in skeletal muscle has been examined, as in other organs, using genetic deletion mouse models.

Skeletal muscle specific deletion of Raptor causes a number of symptoms, including shorter life expectancy, progressively dystrophic muscle with impaired oxidative capacity and increased glycogen stores [40,41]. Activation of mTORC1 signaling in muscle has been examined using mice model with TSC1 deletion specifically in muscle (TSCmKO mice). Although these mice are lean, they develop glucose intolerance and insulin resistance characterized by reduced glucose uptake in the muscle and reduced glycogen and lipid deposition in the liver under high fat diet condition [42,43]. The mechanism of mTORC1 activation induced insulin sensitivity has been examined elaborately. When mTORC1 is activated, S6K1 could directly phosphorylate IRS1 (S307 and S636/S639) and promote its degradation, which subsequently blunts PI3K-AKT activation and its downstream effects such as glucose uptake, glycogen accumulation, etc. [2,3]. 

Interestingly, the other mTORC1 substrate 4E-BP1 in skeletal muscle has a more general effect on systemic metabolism. Overexpression of the mTORC1-nonresponsive form 4E-BP1 in skeletal muscle results in increased energy expenditure, with enhanced respiratory activity both in skeletal muscle and brown fat. Increased PGC-1α activity and the myokine FGF21 expression may partially responsible for the altered metabolic effects in these two tissues [44].

Regulated in development and DNA damage response 1 (REDD1) is an upstream inhibitor of mTORC1 pathway. REDD1 suppresses mTORC1 signaling pathway through de-phosphorylation of AKT and activation of TSC1/TSC2 complex [45]. In turn, mTORC1 activation could stabilize NEDD1 protein as a mTORC1-REDD1 feedback loop [46]. Under the obese or T2DM condition, both mTORC1 signaling and REDD1 protein level are elevated in the skeletal muscle [47,48]. It is proposed that hyper-activation of mTORC1 stabilizes REDD1 protein, which inhibits insulin-induced AKT phosphorylation, attenuating glucose uptake in the skeletal muscle. This may also contribute to hyperglycemia and insulin resistance in T2DM and obese patients [48]. 

It should be noted that mTORC1 activity exerts a significant effect on muscle mass by affecting autophagy process. Sustained activation of mTORC1 in skeletal muscle reduces muscle mass and muscle fiber size in old mice, leading to a late-onset myopathy which is tightly associated with other metabolic pathways [49]. Furthermore, skeletal muscle mass and function are also regulated by motor innervation. mTORC1 is substantially increased in denervated muscle. Mice with mTORC1 activation exhibited increased sensitivity to denervation-induced atrophy. These data reveal that mTORC1 is central to the muscle catabolism and atrophy [50]. The cause and effect relation between glucose metabolism and muscle mass upon mTORC1 activation in skeletal muscle remains to be explored.

The role of mTORC2 in muscle has been evaluated using muscle specific Rictor knockout mice. Similar to mTORC1, mTORC2 regulates insulin-mediated glucose uptake and glucose tolerance. The insulin stimulated phosphorylatin of AKT at Ser473 is significantly reduced in Rictor knockout mice. This alteration is associated with a defect in insulin signaling and the defective glucose transport [51]. Recently Klieinert et al. also found that muscle mTORC2 activity negatively modulates whole body lipid metabolism and intramyocellular triglyceride content through regulating the lipid droplet binding protein Perilipin 3 via FoxO1 [52,53]. 

## 5. mTOR Signaling in Pancreas

mTORC1 has been regarded as a positive regulator of beta cell mass, due to enhancement of beta cell growth and proliferation. The loss-of-function studies of mTOR signaling in vivo have been examined in mice deficient for mTOR or Raptor specifically in beta cells or pancreatic endocrine progenitor cells (mating with PDX1- or Neurog3-cre mice). These mice consistently exhibit reduced beta cells mass, defective postnatal islet development, hypoinsulinemia, and glucose intolerance [54,55,56,57,58]. Conversely, activation of mTORC1 signaling by deletion of TSC1 leads to beta cell hypertrophy and hyperinsulinemia [59]. These observations suggest that mTORC1 is critical for the islet development and function, and consequent glucose homeostasis. mTOR also protects islets from apoptosis by inhibiting the expression of thioredoxin-interacting protein (TXNIP), a potent inducer of β cell death and oxidative stress. TXNIP can be transcriptionally activated by the carbohydrate-response element–binding protein (ChREBP). mTOR physically interacts with ChREBP–Max-like protein complex, consequently suppressing its transcriptional activity on TXNIP [55]. 

However, study of constitutive activation of mTOR reveals a biphasic role of mTOR in the glucose homeostasis. Young mice with beta cell-specific deletion of TSC2 display beta cell hypertrophy, hyperinsulinemia, and improved glucose tolerance. On the other hand, beta cell mass in aging mice is gradually lost due to increased apoptosis, which triggers hyperglycemia [60,61]. Moreover, mTORC1 is aberrantly activated in islets from T2DM patients and diabetic mouse islets, suggesting that sustained hyperactivation of mTORC1 contributes to impaired beta cell function and survival upon the metabolic stress [4]. Chronic hyper-activation of mTORC1 signaling also results in impaired autophagy/mitophagy process and ER stress, evidenced by an accumulation of p62 protein (an indication of impaired autophagic response) and ER stress markers in the older TSC2 knockout beta cells [60]. Consistently, mTOR inhibition protects lipid accumulation, ER stress and beta cell dysfunction under nutrient overload conditions [4,62]. Therefore, mTORC1 activation increases beta cell mass and improves glucose metabolism in the short term, while sustained mTORC1 activation ultimately deteriorates beta cell mass and function, which is reminiscent in type 2 diabetic beta cells.

The molecular mechanisms underlying the detrimental effects of hyper-activation of mTOR signaling in beta cells has been elaborately reviewed recently [63]. Several mechanisms have been proposed: (1) mTORC1 directly phosphorylates IRS1/2 and promotes its degradation, impairs insulin signaling pathway and induces insulin resistance. (2) mTORC1 can phosphorylate and activate Growth-Factor-Bound Protein 10 (Grb10), which disrupts the interaction between IR and IRS1/2, induces IRS2 proteasomal degradation, and ultimately leads to defective insulin signaling pathway [64,65]. (3) mTORC1 can also phosphorylate mTORC2 components, such as Rictor and Sin1, abrogate mTORC2 activity and AKT signaling [66,67]. 

In addition to beta cells, mTORC1 also regulates α cell mass and glucagon secretion. Mice lacking Raptor specifically in α cell have normal α cell mass, but defective α cell maturation and glucagon secretion. FoxA2 is the downstream transcription factor regulating the critical genes responsible for α cell function [68]. Type 2 diabetes patients have elevated glucagon. Glucagon stimulates hepatic digestion of proteins to amino acids. Increased amino acids induce alpha cell hyperplasia by an mTORC1-dependent mechanism [69,70,71]. 

Similar to mTORC1, mTORC2 is also critical for maintaining beta cell mass and glucose homeostasis. Rictor null mice exhibits glucose intolerance caused by a reduction in β-cell mass, beta-cell proliferation, pancreatic insulin content, and glucose-stimulated insulin secretion [72]. In contrast to mTORC1, mTORC2 activity is declined in beta cells under diabetogenic conditions and in human diabetic islets [4].

## 6. mTOR Signaling in Brain

mTOR pathway has been implicated in neural development and neurodegenerative disorders [73]. Hypothalamus is the main structure in the central nervous system (CNS) involved in the control of glucose homeostasis and systemic energy balance. The hypothalamic region comprises several nuclei with distinct functions and serves as a hub which integrates nutrient and hormones signals, regulating the systemic energy balance. Within hypothalamus, mTOR functions as a cellular signaling hub which integrates internal and external cues to control the central or peripheral tissue functions [74].

In the hypothalamus, there are two groups of neurons, orexigenic neuropeptide Y (NPY), and agouti-related protein (AgRP) co-expressing neurons, and anorexigenic proopiomelanocortin (POMC) and cocaine and amphetamine related transcript (CART) co-expressing neurons. mTORC1 activity in the hypothalamus is complex and varies by cell and stimulus type. S6K is expressed in orexigenic NPY and AgRP neurons, as well as POMC neurons, both of which are critically involved in the regulation of energy homeostasis. Increased S6K activity by adenoviral injection of constitutive active form of S6K in the mediobasal hypothalamus (MBH) decreases body weight, food intake, and hypothalamic leptin sensitivity, while increasing thermogenesis during cold challenge. Overexpression of S6K protects high fat feeding induced hyperphagia, fat accumulation and insulin resistance suggesting a critical role of MBH S6K activity in energy homeostasis [75]. Constitutive activation of mTORC1 signaling in the AgRP neurons modulates sympathetic tone to increase BAT thermogenesis and energy expenditure and protects against diet-induced obesity [76]. However, a recent study has shown that specific ablation of S6K1 in POMC or AgRP neurons causes no significant change in food intake and body weight. This discrepancy has been proposed to be possibly caused by high level of adenovirus-mediated gene expression, the local inflammation, the post-operative stress, and/or another group of neurons other than AgRP and POMC neurons involved. In their study, although S6K1 is not required for the hypothalamic regulation of food intake and body weight, it alters the hepatic glucose output (HGP), peripheral lipid metabolism and skeletal muscle insulin sensitivity, suggesting an important role of hypothalamic S6K1 in glucose and energy homeostasis [77]. However, studies using genetic or pharmcological manipulation of mTOR activation in mice demonstrated that mTORC1 hyperactivation in POMC neurons leads to increased body mass and defective neuron activation [78,79]. Therefore, the role of mTORC1 in hypothalamus is elusive and needs further investigation.

DEPTOR is the inhibitor protein of mTOR which is shared by mTORC1 and mTORC2. It is widely expressed in the brain and highly expressed in MBH neurons. Overexpression of DEPTOR specifically in the MBH neurons protects mice from HFD-induced obesity and improves systemic glucose homeostasis due to decreased food intake and elevated oxygen consumption [80]. Since DEPTOR co-localizes with POMC neurons, it is possible that the POMC neurons mediate the effects of hypothalamic DEPTOR overexpression. Unexpectedly, none of these phenotypes is reproduced in the mice with POMC–specific overexpression of mTORC1, suggesting that other neuronal populations in the MBH are responsible for the energy and glucose metabolism control [81]. It should be noted that the mTORC1 signaling is implicated in the neuronal growth/migration and synaptic plasticity. Brain somatic activating mutations in components of the PI3K-AKT-TSC1/2-mTOR pathway have also been identified in the epileptogenic neurodevelopmental disease, focal cortical dysplasia (FCD) type II [82,83,84]. The critical developmental defects may profoundly impact the systemic metabolism.

The implication of mTORC2 in central regulation of energy balance is much less well defined. Kocalis et al. found that mice lacking Rictor in nestin-positive neural cells exhibits increased fat mass and adiposity, as well as glucose intolerance. Moreover, they examined mice with Rictor deletion in POMC and AgRP neurons. Loss of Rictor in POMC neurons reproduces most of phenotypes such as hyperphagia, obesity, and glucose intolerance, while loss of Rictor in AgRP neurons has no significant effects on energy homeostasis [85]. Since mTORC2 signaling is also implicated in the neural development [86], it is possible that the energy dys-homeostasis is caused by defective neuron morphology and function.

## 7. Therapeutic Potential of Targeting mTOR Signaling Pathway

Since mTORC1 has been aberrantly increased in the diabetes or metabolic stressed conditions, targeting mTORC1 signaling pathway represents a potential treatment for metabolic dys-regulation. Rapamycin is the well-known, classical mTORC1 inhibitor. It forms a protein complex with FKBP12 or FKBP51 and inhibits mTORC1 function. Rapamycin treatment exerts significant effects on systemic metabolism affecting multiple organs [87]. Acute treatment of rapamycin enhances insulin secretion and prevents nutrient-induced insulin resistance. However, a number of studies have demonstrated that chronic rapamycin treatment induces detrimental effects on metabolic profiling, including reduced beta cell mass and function, increased hepatic gluconeogenesis, and impaired insulin sensitivity [37,88,89,90,91]. 

Recently, one interesting study has compared the effects of rapamycin treatment on different diabetic mouse models and unexpectedly demonstrated that rapamycin improves insulin sensitivity and reduces hyperinsulinemia better in mice with lower pancreatic insulin content. It has thus been proposed that the beneficial or detrimental effects of rapamycin treatment are determined by the pancreatic insulin contents and pancreas biology [92].

Another explanation for the detrimental effects of rapamycin is the “off-target” effect on mTORC2 disruption. Chronic administration of rapamycin also disrupts mTORC2 complex and impairs mTORC2 signaling, which is required for the insulin-mediated suppression of hepatic gluconeogenesis [37]. Rapamycin also causes mTORC2-dependent insulin resistance in C2C12 myotubes [93]. These findings prompt the development of specific inhibitors of mTORC1 which might provide beneficial effects on health and longevity avoiding of the detrimental effects on systemic metabolism.

In order to inhibit mTORC1 signaling pathway more specifically, an inhibitor of S6K1, PF-4708671 has been generated and used for delineating S6K1-specific roles downstream of mTOR [94]. Shum et al. have compared the effects of glucose metabolism using rapamycin and PF-4708671 in vitro and in vivo. In contrast to the adverse effects associated with chronic rapamycin treatment, S6K1 inhibition with PF-4708671 improves glucose tolerance with increased AKT phosphorylation in both cellular and high fat diet induced obese mouse models [95]. Therefore, specific S6K1 blockade is a promising pharmacological approach to improve metabolic homeostasis in obese or diabetic individuals.

## 8. Concluding Remarks

During the past few decades, our knowledge on mTOR regulatory mechanism in these key metabolic organs at the molecular, cellular, and organismal level has been emerging (Table 1). Rapamycin and several other mTOR targeting drugs have been used for cancers and immuno-suppressive therapies. However, their side effects leading to dys-regulated metabolic profiling limit their clinical use. More basic and clinical studies are required to better understand the beneficial and side effects of mTOR inhibiting strategy against metabolic disorders. In addition, a comprehensive understanding of mTORC2 pathway, identification of new mTOR signaling substrates and molecular mechanisms would pave the way for developing more specific treatment in the future.

## Figures and Tables

**Table 1:** The effects of altered mTOR signaling on glucose and lipid metabolism in metabolic tissues.

	mTORC1	mTORC2
Adipose tissue	Normal adipose tissue growth [16]; BAT-WAT phenotypic switch [18,19,20]	Regulate fat and whole body organ size [21]; systemic glucose and lipid metabolism [22]; BAT differentiation [25]
Liver	Suppress ketogenesis in response to fasting [27]; promote lipogenesis [28,29]	Regulate constitutive gluconeogenesis, increase glycolysis and lipogenesis [35,36]
Muscle	Glucose intolerance and insulin resistance, hypertrophy [42,43]	Promote glucose uptake and improve insulin signaling [51]; negatively modualtes systemic lipid metabolism and intramyocellular triglycerid content [52,53]
Pancreas	Promote beta cell growth and proliferation [54,55,56,57,58]; improved glucose tolerance in short term, deteriorates beta cell mass and function in long term [60,61]; maintain α cell maturation and glucagon secretion [68]	Maintaining beta cell mass and glucose homeostasis [72]
Brain	Regulate the hepatic glucose output, peripheral lipid metabolism and skeletal muscle insulin sensitivity [75,76,77]	Regulate fat mass and adiposity, and glucose tolerance [85]

## References

[1] Saxton R.A., Sabatini D.M. (2017). mTOR Signaling in Growth, Metabolism, and Disease. Cell.

[2] Um S.H., Frigerio F., Watanabe M., Picard F., Joaquin M., Sticker M., Fumagalli S., Allegrini P.R., Kozma S.C., Auwerx J. (2004). Absence of S6K1 protects against age- and diet-induced obesity while enhancing insulin sensitivity. Nature.

[3] Laplante M., Sabatini D.M. (2012). mTOR signaling in growth control and disease. Cell.

[4] Yuan T., Rafizadeh S., Gorrepati K.D.D., Lupse B., Oberholzer J., Maedler K., Ardestani A. (2017). Reciprocal regulation of mTOR complexes in pancreatic islets from humans with type 2 diabetes. Diabetologia.

[5] Sancak Y., Bar-Peled L., Zoncu R., Markhard A.L., Nada S., Sabatini D.M. (2010). Ragulator-Rag Complex Targets mTORC1 to the Lysosomal Surface and Is Necessary for Its Activation by Amino Acids. Cell.

[6] Kim E., Goraksha-Hicks P., Li L., Neufeld T.P., Guan K.-L. (2008). Regulation of TORC1 by Rag GTPases in nutrient response. Nat. Cell Biol..

[7] Inoki K., Zhu T., Guan K.L. (2003). TSC2 mediates cellular energy response to control cell growth and survival. Cell.

[8] Cornu M., Albert V., Hall M.N. (2013). mTOR in aging, metabolism, and cancer. Curr. Opin. Genet. Dev..

[9] Ben-Sahra I., Hoxhaj G., Ricoult S.J.H., Asara J.M., Manning B.D. (2016). mTORC1 induces purine synthesis through control of the mitochondrial tetrahydrofolate cycle. Science.

[10] Düvel K., Yecies J.L., Menon S., Raman P., Lipovsky A.I., Souza A.L., Triantafellow E., Ma Q., Gorski R., Cleaver S. (2010). Activation of a Metabolic Gene Regulatory Network Downstream of mTOR Complex 1. Mol. Cell.

[11] Peterson T.R., Sengupta S.S., Harris T.E., Carmack A.E., Kang S.A., Balderas E., Guertin D.A., Madden K.L., Carpenter A.E., Finck B.N. (2011). mTOR Complex 1 Regulates Lipin 1 Localization to Control the SREBP Pathway. Cell.

[12] Kim J., Kundu M., Viollet B., Guan K.-L. (2011). AMPK and mTOR regulate autophagy through direct phosphorylation of Ulk1. Nat. Cell Biol..

[13] Settembre C., Zoncu R., Medina D.L., Vetrini F., Erdin S., Erdin S., Huynh T., Ferron M., Karsenty G., Vellard M.C. (2012). A lysosome-to-nucleus signalling mechanism senses and regulates the lysosome via mTOR and TFEB. EMBO J..

[14] Zhao J., Zhai B., Gygi S.P., Goldberg A.L. (2015). mTOR inhibition activates overall protein degradation by the ubiquitin proteasome system as well as by autophagy. Proc. Natl. Acad. Sci. USA.

[15] Rousseau A., Bertolotti A. (2016). An evolutionarily conserved pathway controls proteasome homeostasis. Nature.

[16] Shan T., Zhang P., Jiang Q., Xiong Y., Wang Y., Kuang S. (2016). Adipocyte-specific deletion of mTOR inhibits adipose tissue development and causes insulin resistance in mice. Diabetologia.

[17] Arif A., Terenzi F., Potdar A.A., Jia J., Sacks J., China A., Halawani D., Vasu K., Li X.X., Brown J.M. (2017). EPRS is a critical mTORC1-S6K1 effector that influences adiposity in mice. Nature.

[18] Xiang X., Lan H., Tang H., Yuan F., Xu Y., Zhao J., Li Y., Zhang W. (2015). Tuberous Sclerosis Complex 1–Mechanistic Target of Rapamycin Complex 1 Signaling Determines Brown-to-White Adipocyte Phenotypic Switch. Diabetes.

[19] Liu D.X., Bordicchia M., Zhang C.Y., Fang H.F., Wei W., Li J.L., Guilherme A., Guntur K., Czech M.P., Collins S. (2016). Activation of mTORC1 is essential for beta-adrenergic stimulation of adipose browning. J. Clin. Investig..

[20] Tran C.M., Mukherjee S., Ye L., Frederick D.W., Kissig M., Davis J.G., Lamming D.W., Seale P., Baur J.A. (2016). Rapamycin Blocks Induction of the Thermogenic Program in White Adipose Tissue. Diabetes.

[21] Cybulski N., Polak P., Auwerx J., Rüegg M.A., Hall M.N. (2009). mTOR complex 2 in adipose tissue negatively controls whole-body growth. Proc. Natl. Acad. Sci. USA.

[22] Kumar A., Lawrence J.C., Jung D.Y., Ko H.J., Keller S.R., Kim J.K., Magnuson M.A., Harris T.E. (2010). Fat cell-specific ablation of rictor in mice impairs insulin-regulated fat cell and whole-body glucose and lipid metabolism. Diabetes.

[23] Yao Y., Suraokar M., Darnay B.G., Hollier B.G., Shaiken T.E., Asano T., Chen C.-H., Chang B.H.-J., Lu Y., Mills G.B. (2013). BSTA Promotes mTORC2-Mediated Phosphorylation of Akt1 to Suppress Expression of FoxC2 and Stimulate Adipocyte Differentiation. Sci. Signal..

[24] Tang Y.F., Wallace M., Sanchez-Gurmaches J., Hsiao W.Y., Li H.W., Lee P.L., Vernia S., Metallo C.M., Guertin D.A. (2016). Adipose tissue mTORC2 regulates ChREBP-driven de novo lipogenesis and hepatic glucose metabolism. Nat. Commun..

[25] Hung C.-M., Calejman C.M., Sanchez-Gurmaches J., Li H., Clish C.B., Hettmer S., Wagers A.J., Guertin D.A. (2014). Rictor/mTORC2 loss in the Myf5 lineage reprograms brown fat metabolism and protects mice against obesity and metabolic disease. Cell Rep..

[26] Albert V., Svensson K., Shimobayashi M., Colombi M., Muñoz S., Jimenez V., Handschin C., Bosch F., Hall M.N. (2016). mTORC2 sustains thermogenesis via Akt-induced glucose uptake and glycolysis in brown adipose tissue. EMBO Mol. Med..

[27] Sengupta S., Peterson T.R., Laplante M., Oh S., Sabatini D.M. (2010). mTORC1 controls fasting-induced ketogenesis and its modulation by ageing. Nature.

[28] Li Z., Xu G., Qin Y., Zhang C., Tang H., Yin Y., Xiang X., Li Y., Zhao J., Mulholland M. (2014). Ghrelin promotes hepatic lipogenesis by activation of mTOR-PPARγ signaling pathway. Proc. Natl. Acad. Sci. USA.

[29] Han J.B., Li E.W., Chen L.Q., Zhang Y.Y., Wei F.C., Liu J.Y., Deng H.T., Wang Y.G. (2015). The CREB coactivator CRTC2 controls hepatic lipid metabolism by regulating SREBP1. Nature.

[30] Kim K., Qiang L., Hayden M.S., Sparling D.P., Purcell N.H., Pajvani U.B. (2016). mTORC1-independent Raptor prevents hepatic steatosis by stabilizing PHLPP2. Nat. Commun..

[31] Cornu M., Oppliger W., Albert V., Robitaille A.M., Trapani F., Quagliata L., Fuhrer T., Sauer U., Terracciano L., Hall M.N. (2014). Hepatic mTORC1 controls locomotor activity, body temperature, and lipid metabolism through FGF21. Proc. Natl. Acad. Sci. USA.

[32] Lee J.H., Budanov A.V., Talukdar S., Park E.J., Park H.L., Park H.W., Bandyopadhyay G., Li N., Aghajan M., Jang I. (2012). Maintenance of metabolic homeostasis by Sestrin2 and Sestrin3. Cell Metab..

[33] Park H.W., Park H., Ro S.H., Jang I., Semple I.A., Kim D.N., Kim M., Nam M., Zhang D., Yin L. (2014). Hepatoprotective role of Sestrin2 against chronic ER stress. Nat. Commun..

[34] Howell J.J., Hellberg K., Turner M., Talbott G., Kolar M.J., Ross D.S., Hoxhaj G., Saghatelian A., Shaw R.J., Manning B.D. (2017). Metformin Inhibits Hepatic mTORC1 Signaling via Dose-Dependent Mechanisms Involving AMPK and the TSC Complex. Cell Metab..

[35] Hagiwara A., Cornu M., Cybulski N., Polak P., Betz C., Trapani F., Terracciano L., Heim M.H., Rüegg M.A., Hall M.N. (2012). Hepatic mTORC2 Activates Glycolysis and Lipogenesis through Akt, Glucokinase, and SREBP1c. Cell Metab..

[36] Yuan M., Pino E., Wu L., Kacergis M., Soukas A.A. (2012). Identification of Akt-independent Regulation of Hepatic Lipogenesis by Mammalian Target of Rapamycin (mTOR) Complex 2. J. Biol. Chem..

[37] Lamming D.W., Ye L., Katajisto P., Goncalves M.D., Saitoh M., Stevens D.M., Davis J.G., Salmon A.B., Richardson A., Ahima R.S. (2012). Rapamycin-Induced Insulin Resistance Is Mediated by mTORC2 Loss and Uncoupled from Longevity. Science.

[38] Tao R., Xiong X., Liangpunsakul S., Dong X.C. (2015). Sestrin 3 Protein Enhances Hepatic Insulin Sensitivity by Direct Activation of the mTORC2-Akt Signaling. Diabetes.

[39] Javary J., Allain-Courtois N., Saucisse N., Costet P., Heraud C., Benhamed F., Pierre R., Bure C., Pallares-Lupon N., Do Cruzeiro M. (2017). Liver Reptin/RUVBL2 controls glucose and lipid metabolism with opposite actions on mTORC1 and mTORC2 signalling. Gut.

[40] Bentzinger C.F., Romanino K., Cloëtta D., Lin S., Mascarenhas J.B., Oliveri F., Xia J., Casanova E., Costa C.F., Brink M. (2008). Skeletal Muscle-Specific Ablation of raptor, but Not of rictor, Causes Metabolic Changes and Results in Muscle Dystrophy. Cell Metab..

[41] Lopez R.J., Mosca B., Treves S., Maj M., Bergamelli L., Calderon J.C., Bentzinger C.F., Romanino K., Hall M.N., Ruegg M.A. (2015). Raptor ablation in skeletal muscle decreases Cav1.1 expression and affects the function of the excitation-contraction coupling supramolecular complex. Biochem. J..

[42] Guridi M., Kupr B., Romanino K., Lin S., Falcetta D., Tintignac L., Rüegg M.A. (2016). Alterations to mTORC1 signaling in the skeletal muscle differentially affect whole-body metabolism. Skeletal Muscle.

[43] Guridi M., Tintignac L.A., Lin S., Kupr B., Castets P., Rüegg M.A. (2015). Activation of mTORC1 in skeletal muscle regulates whole-body metabolism through FGF21. Sci. Signal..

[44] Tsai S., Sitzmann J.M., Dastidar S.G., Rodriguez A.A., Vu S.L., McDonald C.E., Academia E.C., O’Leary M.N., Ashe T.D., La Spada A.R. (2015). Muscle-specific 4E-BP1 signaling activation improves metabolic parameters during aging and obesity. J. Clin. Investig..

[45] Lipina C., Hundal H.S. (2016). Is REDD1 a Metabolic Eminence Grise?. Trends Endocrinol. Metab. TEM.

[46] Tan C.Y., Hagen T. (2013). mTORC1 dependent regulation of REDD1 protein stability. PLoS ONE.

[47] Williamson D.L., Li Z., Tuder R.M., Feinstein E., Kimball S.R., Dungan C.M. (2014). Altered nutrient response of mTORC1 as a result of changes in REDD1 expression: Effect of obesity vs. REDD1 deficiency. J. Appl. Physiol..

[48] Williamson D.L., Dungan C.M., Mahmoud A.M., Mey J.T., Blackburn B.K., Haus J.M. (2015). Aberrant REDD1-mTORC1 responses to insulin in skeletal muscle from Type 2 diabetics. Am. J. Physiol. Regul. Integr. Comp. Physiol..

[49] Castets P., Lin S., Rion N., Di Fulvio S., Romanino K., Guridi M., Frank S., Tintignac L.A., Sinnreich M., Rüegg M.A. (2013). Sustained Activation of mTORC1 in Skeletal Muscle Inhibits Constitutive and Starvation-Induced Autophagy and Causes a Severe, Late-Onset Myopathy. Cell Metab..

[50] Tang H., Inoki K., Lee M., Wright E., Khuong A., Khuong A., Sugiarto S., Garner M., Paik J., DePinho R.A. (2014). mTORC1 Promotes Denervation-Induced Muscle Atrophy Through a Mechanism Involving the Activation of FoxO and E3 Ubiquitin Ligases. Sci. Signal..

[51] Kumar A., Harris T.E., Keller S.R., Choi K.M., Magnuson M.A., Lawrence J.C. (2008). Muscle-specific deletion of rictor impairs insulin-stimulated glucose transport and enhances Basal glycogen synthase activity. Mol. Cell Biol..

[52] Kleinert M., Sylow L., Fazakerley D.J., Krycer J.R., Thomas K.C., Oxboll A.J., Jordy A.B., Jensen T.E., Yang G., Schjerling P. (2014). Acute mTOR inhibition induces insulin resistance and alters substrate utilization in vivo. Mol. Metab..

[53] Kleinert M., Parker B.L., Chaudhuri R., Fazakerley D.J., Serup A., Thomas K.C., Krycer J.R., Sylow L., Fritzen A.M., Hoffman N.J. (2016). mTORC2 and AMPK differentially regulate muscle triglyceride content via Perilipin 3. Mol. Metab..

[54] Blandino-Rosano M., Barbaresso R., Jimenez-Palomares M., Bozadjieva N., Werneck-de-Castro J.P., Hatanaka M., Mirmira R.G., Sonenberg N., Liu M., Ruegg M.A. (2017). Loss of mTORC1 signalling impairs beta-cell homeostasis and insulin processing. Nat. Commun..

[55] Chau G.C., Im D.U., Kang T.M., Bae J.M., Kim W., Pyo S., Moon E.-Y., Um S.H. (2017). mTOR controls ChREBP transcriptional activity and pancreatic β cell survival under diabetic stress. J. Cell Biol..

[56] Elghazi L., Blandino-Rosano M., Alejandro E., Cras-Méneur C., Bernal-Mizrachi E. (2017). Role of nutrients and mTOR signaling in the regulation of pancreatic progenitors development. Mol. Metab..

[57] Ni Q., Gu Y., Xie Y., Yin Q., Zhang H., Nie A., Li W., Wang Y., Ning G., Wang W. (2017). Raptor regulates functional maturation of murine beta cells. Nat. Commun..

[58] Sinagoga K.L., Stone W.J., Schiesser J.V., Schweitzer J.I., Sampson L., Zheng Y., Wells J.M. (2017). Distinct roles for the mTOR pathway in postnatal morphogenesis, maturation and function of pancreatic islets. Development.

[59] Ding L., Yin Y., Han L., Li Y., Zhao J., Zhang W. (2017). TSC1-mTOR signaling determines the differentiation of islet cells. J. Endocrinol..

[60] Bartolome A., Kimura-Koyanagi M., Asahara S.I., Guillen C., Inoue H., Teruyama K., Shimizu S., Kanno A., Garcia-Aguilar A., Koike M. (2014). Pancreatic beta-Cell Failure Mediated by mTORC1 Hyperactivity and Autophagic Impairment. Diabetes.

[61] Rachdi L., Balcazar N., Osorio-Duque F., Elghazi L., Weiss A., Gould A., Chang-Chen K.J., Gambello M.J., Bernal-Mizrachi E. (2008). Disruption of Tsc2 in pancreatic beta cells induces beta cell mass expansion and improved glucose tolerance in a TORC1-dependent manner. Proc. Natl. Acad. Sci. USA.

[62] Varshney R., Varshney R., Mishra R., Roy P. (2018). Kaempferol alleviates palmitic acid-induced lipid stores, endoplasmic reticulum stress and pancreatic β-cell dysfunction through AMPK/mTOR-mediated lipophagy. J. Nutr. Biochem..

[63] Ardestani A., Lupse B., Kido Y., Leibowitz G., Maedler K. (2018). mTORC1 Signaling: A Double-Edged Sword in Diabetic beta Cells. Cell Metab..

[64] Warren K.J., Fang X., Gowda N.M., Thompson J.J., Heller N.M. (2016). The TORC1-activated Proteins, p70S6K and GRB10, Regulate IL-4 Signaling and M2 Macrophage Polarization by Modulating Phosphorylation of Insulin Receptor Substrate-2. J. Biol. Chem..

[65] Wick K.R., Werner E.D., Langlais P., Ramos F.J., Dong L.Q., Shoelson S.E., Liu F. (2003). Grb10 Inhibits Insulin-stimulated Insulin Receptor Substrate (IRS)-Phosphatidylinositol 3-Kinase/Akt Signaling Pathway by Disrupting the Association of IRS-1/IRS-2 with the Insulin Receptor. J. Biol. Chem..

[66] Julien L.-A., Carriere A., Moreau J., Roux P.P. (2010). mTORC1-activated S6K1 phosphorylates Rictor on threonine 1135 and regulates mTORC2 signaling. Mol. Cell. Biol..

[67] Liu P., Gan W., Inuzuka H., Lazorchak A.S., Gao D., Arojo O., Liu D., Wan L., Zhai B., Yu Y. (2013). Sin1 phosphorylation impairs mTORC2 complex integrity and inhibits downstream Akt signalling to suppress tumorigenesis. Nat. Cell Biol..

[68] Bozadjieva N., Blandino-Rosano M., Chase J., Dai X.-Q., Cummings K., Gimeno J., Dean D., Powers A.C., Gittes G.K., Rüegg M.A. (2017). Loss of mTORC1 signaling alters pancreatic α cell mass and impairs glucagon secretion. J. Clin. Investig..

[69] Dean E.D., Li M., Prasad N., Wisniewski S.N., Von Deylen A., Spaeth J., Maddison L., Botros A., Sedgeman L.R., Bozadjieva N. (2017). Interrupted Glucagon Signaling Reveals Hepatic α Cell Axis and Role for L-Glutamine in α Cell Proliferation. Cell Metab..

[70] Kim J., Okamoto H., Huang Z., Anguiano G., Chen S., Liu Q., Cavino K., Xin Y., Na E., Hamid R. (2017). Amino Acid Transporter Slc38a5 Controls Glucagon Receptor Inhibition-Induced Pancreatic α Cell Hyperplasia in Mice. Cell Metab..

[71] Solloway M.J., Madjidi A., Gu C., Eastham-Anderson J., Clarke H.J., Kljavin N., Zavala-Solorio J., Kates L., Friedman B., Brauer M. (2015). Glucagon Couples Hepatic Amino Acid Catabolism to mTOR-Dependent Regulation of α-Cell Mass. Cell Rep..

[72] Gu Y., Lindner J., Kumar A., Yuan W., Magnuson M.A. (2011). Rictor/mTORC2 Is Essential for Maintaining a Balance Between β-Cell Proliferation and Cell Size. Diabetes.

[73] Bockaert J., Marin P. (2015). mTOR in Brain Physiology and Pathologies. Physiol. Rev..

[74] Hu F., Xu Y., Liu F. (2016). Hypothalamic roles of mTOR complex I: Integration of nutrient and hormone signals to regulate energy homeostasis. Am. J. Physiol.-Endocrinol. Metab..

[75] Blouet C., Ono H., Schwartz G.J. (2008). Mediobasal Hypothalamic p70 S6 Kinase 1 Modulates the Control of Energy Homeostasis. Cell Metab..

[76] Burke L.K., Darwish T., Cavanaugh A.R., Virtue S., Roth E., Morro J., Liu S.M., Xia J., Dalley J.W., Burling K. (2017). mTORC1 in AGRP neurons integrates exteroceptive and interoceptive food-related cues in the modulation of adaptive energy expenditure in mice. eLife.

[77] Smith M.A., Katsouri L., Irvine E.E., Hankir M.K., Pedroni S.M.A., Voshol P.J., Gordon M.W., Choudhury A.I., Woods A., Vidal-Puig A. (2015). Ribosomal S6K1 in POMC and AgRP Neurons Regulates Glucose Homeostasis but Not Feeding Behavior in Mice. Cell Rep..

[78] Mori H., Inoki K., Munzberg H., Opland D., Faouzi M., Villanueva E.C., Ikenoue T., Kwiatkowski D., MacDougald O.A., Myers M.G. (2009). Critical role for hypothalamic mTOR activity in energy balance. Cell Metab..

[79] Yang S.B., Tien A.C., Boddupalli G., Xu A.W., Jan Y.N., Jan L.Y. (2012). Rapamycin ameliorates age-dependent obesity associated with increased mTOR signaling in hypothalamic POMC neurons. Neuron.

[80] Caron A., Labbé S.M., Lanfray D., Blanchard P.-G., Villot R., Roy C., Sabatini D.M., Richard D., Laplante M. (2016). Mediobasal hypothalamic overexpression of DEPTOR protects against high-fat diet-induced obesity. Mol. Metab..

[81] Caron A., Labbe S.M., Mouchiroud M., Huard R., Lanfray D., Richard D., Laplante M. (2016). DEPTOR in POMC neurons affects liver metabolism but is dispensable for the regulation of energy balance. Am. J. Physiol.-Regul. Integr. Comp. Physiol..

[82] Park A.H., Park E.K., Cho Y.W., Kim S., Kim H.M., Kim J.A., Kim J., Rhee H., Kang S.G., Kim H.D. (2015). Brain somatic mutations in MTOR cause focal cortical dysplasia type II leading to intractable epilepsy. Nat. Med..

[83] Lim J.S., Gopalappa R., Kim S.H., Ramakrishna S., Lee M., Kim W.I., Kim J., Park S.M., Lee J., Oh J.H. (2017). Somatic Mutations in TSC1 and TSC2 Cause Focal Cortical Dysplasia. Am. J. Hum. Genet..

[84] Park S.M., Lim J.S., Ramakrishina S., Kim S.H., Kim W.K., Lee J., Kang H.C., Reiter J.F., Kim D.S., Kim H.H. (2018). Brain Somatic Mutations in MTOR Disrupt Neuronal Ciliogenesis, Leading to Focal Cortical Dyslamination. Neuron.

[85] Kocalis H.E., Hagan S.L., George L., Turney M.K., Siuta M.A., Laryea G.N., Morris L.C., Muglia L.J., Printz R.L., Stanwood G.D. (2014). Rictor/mTORC2 facilitates central regulation of energy and glucose homeostasis. Mol. Metab..

[86] Thomanetz V., Angliker N., Cloëtta D., Lustenberger R.M., Schweighauser M., Oliveri F., Suzuki N., Rüegg M.A. (2013). Ablation of the mTORC2 component rictor in brain or Purkinje cells affects size and neuron morphology. J. Cell Biol..

[87] Harrison D.E., Strong R., Sharp Z.D., Nelson J.F., Astle C.M., Flurkey K., Nadon N.L., Wilkinson J.E., Frenkel K., Carter C.S. (2009). Rapamycin fed late in life extends lifespan in genetically heterogeneous mice. Nature.

[88] Barlow A.D., Nicholson M.L., Herbert T.P. (2013). Evidence for Rapamycin Toxicity in Pancreatic β-Cells and a Review of the Underlying Molecular Mechanisms. Diabetes.

[89] Deblon N., Bourgoin L., Veyrat-Durebex C., Peyrou M., Vinciguerra M., Caillon A., Maeder C., Fournier M., Montet X., Rohner-Jeanrenaud F. (2012). Chronic mTOR inhibition by rapamycin induces muscle insulin resistance despite weight loss in rats. Br. J. Pharmacol..

[90] Houde V.P., Brule S., Festuccia W.T., Blanchard P.G., Bellmann K., Deshaies Y., Marette A. (2010). Chronic Rapamycin Treatment Causes Glucose Intolerance and Hyperlipidemia by Upregulating Hepatic Gluconeogenesis and Impairing Lipid Deposition in Adipose Tissue. Diabetes.

[91] Pereira M.J., Palming J., Rizell M., Aureliano M., Carvalho E., Svensson M.K., Eriksson J.W. (2012). mTOR inhibition with rapamycin causes impaired insulin signalling and glucose uptake in human subcutaneous and omental adipocytes. Mol. Cell. Endocrinol..

[92] Reifsnyder P.C., Flurkey K., Te A., Harrison D.E. (2016). Rapamycin treatment benefits glucose metabolism in mouse models of type 2 diabetes. Aging.

[93] Ye L., Varamini B., Lamming D., Sabatini D., Baur J. (2012). Rapamycin has a biphasic effect on insulin sensitivity in C2C12 myotubes due to sequential disruption of mTORC1 and mTORC2. Front. Genet..

[94] Pearce L.R., Alton G.R., Richter D.T., Kath J.C., Lingardo L., Chapman J., Hwang C., Alessi D.R. (2010). Characterization of PF-4708671, a novel and highly specific inhibitor of p70 ribosomal S6 kinase (S6K1). Biochem. J..

[95] Shum M., Bellmann K., St-Pierre P., Marette A. (2016). Pharmacological inhibition of S6K1 increases glucose metabolism and Akt signalling in vitro and in diet-induced obese mice. Diabetologia.

